# Association of B7H3 with HIF-1α nuclear expression indicates poor prognosis and therapeutic potential in gastric cancer

**DOI:** 10.3389/fonc.2026.1744341

**Published:** 2026-02-03

**Authors:** Mengsi Li, Tianwei Guo, Ziyi Wang, Xue Liu, Lingchuan Guo, Lei Cao

**Affiliations:** 1 Department of Pathology, The First Affiliated Hospital of Soochow University, Suzhou, Jiangsu, China; 2 Department of Pathology, Changshu Hospital Affiliated to Nanjing University of Chinese Medicine, Changshu, Jiangsu, China; 3 Jiangsu Institute of Clinical Immunology, The First Affiliated Hospital of Soochow University, Suzhou, Jiangsu, China; 4 Jiangsu Key Laboratory of Clinical Immunology, Soochow University, Suzhou, Jiangsu, China; 5 Jiangsu Key Laboratory of Gastrointestinal Tumor Immunology, The First Affiliated Hospital of Soochow University, Suzhou, Jiangsu, China

**Keywords:** B7 homolog 3 (B7H3), gastric cancer, hypoxia-inducible factor-1α (HIF-1α), prognostic biomarker, therapeutic target for gastric cancer

## Abstract

B7H3 (B7 homolog 3) is an important immune checkpoint molecule in the B7-CD28 family, and substantial evidence indicates that it promotes tumor growth, invasion, and metastasis. The imbalance between oxygen supply and consumption in the tumor microenvironment induces hypoxia, which activates hypoxia-inducible factor-1α (HIF-1α) signaling. HIF-1α plays a critical role in tumor growth, metastasis, and immune evasion. However, the interaction between HIF-1α and B7H3 in gastric cancer remains unclear. In this study, we explored the expression characteristics and correlation of B7H3 and HIF-1α in large gastric cancer samples using bioinformatics and immunohistochemical methods. The results show that B7H3 and HIF-1α mRNA are significantly upregulated in gastric cancer, with a strong positive correlation between their expressions. In gastric cancer tissues, no significant correlation was found between B7H3 and HIF-1α expression (r_S_ = 0.070, *P* = 0.257), whereas high B7H3 expression demonstrated a moderate positive correlation with nuclear expression of HIF-1α (r_S_ = 0.141, *P* = 0.021). Furthermore, we found that nuclear expression of HIF-1α was closely associated with poor prognosis in gastric cancer patients (*P* < 0.001) and could serve as an independent risk factor. Notably, knockdown of B7H3 in gastric cancer cells significantly inhibited both the expression and nuclear localization of HIF-1α, whereas overexpression of B7H3 markedly promoted HIF-1α expression. In conclusion, the combination of B7H3 and HIF-1α may serve as a novel prognostic biomarker for gastric cancer and also holds potential as a therapeutic target for its treatment.

## 1 Introduction

Gastric accounts for approximately 10.4% of cancer-related deaths worldwide, making it the fifth most common cancer globally (1). Due to its lack of distinct clinical symptoms in the early stages, gastric cancer is often only first diagnosed at an advanced stage (2). Currently, surgical treatment is the primary method for treating gastric cancer, but the overall five-year survival rate remains below 25% (3). In recent years, immunotherapy and targeted therapy have made significant progress in the treatment of gastric cancer, however. Among these, the blockade of the PD-1/PD-L1 pathway is a promising approach, with studies showing that anti-PD-1 immunotherapy can induce durable antitumor responses and long-term remission (4). However, PD-1 monotherapy still has limitations to therapeutic efficacy for certain gastric cancer populations (5). Therefore, there is an urgent need to develop new immune checkpoints and biomarkers.

B7 homolog 3 protein (B7H3), also known as CD276, is a member of the B7 molecule family and belongs to the B7 immunoglobulin superfamily as a type I transmembrane protein. Like PD-1, B7H3 possesses both immune-dependent and immune-independent functions (6, 7). Several studies have found that B7H3 is overexpressed in various types of gastric malignant tumors and is often associated with poor clinical prognosis in patients with gastric cancer (8, 9). Furthermore, recent studies have shown that monoclonal antibody therapy targeting B7H3 or B7H3-directed CAR-T therapy can achieve clinical success in cancer treatment (10–12).

As tumors progress, various factors contribute to their immune tolerance by transforming the anti-tumor microenvironment into a pro-tumor microenvironment. For example, the rapid and uncontrolled proliferation of tumors limits the availability of oxygen, making hypoxia a typical microenvironmental feature of nearly all solid tumors, and this can lead to the generation of an immunosuppressive tumor microenvironment (13). Hypoxia also activates the hypoxia-inducible factor (HIF) family, which regulates gene expression within tumor cells and immune cells in the tumor microenvironment, influencing tumor progression and resistance (14, 15). Hypoxia-inducible factor-1 alpha (HIF-1α) is the primary regulatory protein that responds to changes in cellular oxygen levels, and studies have shown that hypoxia can induce high expression of HIF-1α (16). Several studies have demonstrated that HIF-1α can be stably expressed in gastric cancer (17), promoting gastric cancer cell proliferation (18), and participating in gastric cancer metastasis (19), angiogenesis (20), and resistance (21). Therefore, HIF-1α may serve as a gastric cancer-specific biomarker and a promising therapeutic target.

Currently, studies have shown that combination therapy using HIF-1α inhibitors and PD-L1 blockers can enhance the immune system of patients with cancer (22). Additionally, research on oral squamous cell carcinoma (23) and lung cancer (24) has shown that B7H3 can promote the expression of HIF-1α. Therefore, as a member of the same family as PD-1, the combined treatment of B7H3 with HIF-1α holds significant promise for clinical application. To date, however, there have been no studies of the relationship between the two, nor have there been studies of their combined impact on clinical outcomes in gastric cancer patients. Therefore, this study employed bioinformatics approaches to analyze both the correlation between B7H3 and HIF-1α mRNA expression in gastric cancer and their collective association with patient survival prognosis. In parallel, we assessed the relationship between B7H3 and HIF-1α protein expression levels through comprehensive detection of these proteins in gastric cancer tissues and cell line models.

## 2 Materials and methods

### 2.1 Bioinformatics data source and analysis

Bioinformatics analysis data were obtained from the GEPIA database (http://gepia.cancer-pku.cn/), and TCGA-STAD expression data, including count data and sample survival information, were retrieved from the UCSC database. The mRNA expression data of B7H3 and HIF-1α in TCGA-STAD were also extracted from the UCSC database (375 gastric cancer samples and 32 adjacent noncancerous tissues). The Wilcoxon test was used to analyze the differences in B7H3 and HIF-1α mRNA expression between gastric cancer and adjacent normal tissues, as well as their correlation. The ggplot2 package was employed for graphical visualization.

### 2.2 Clinical data

A total of 268 gastric cancer patient samples were collected for this study. Only histologically confirmed cases were included, while cases with incomplete clinical data were excluded. Among the 268 patients, 210 were male and 58 were female, with ages ranging from 33 to 88 years (mean age: 64.5 years). These patients were diagnosed with gastric cancer in 2011 by two qualified pathologists from the Department of Pathology at the First Affiliated Hospital of Soochow University, Suzhou, China. All gastric cancer specimens were obtained from surgical resections, fixed in formalin, and embedded in paraffin. Clinical and pathological data, including gender, age, tumor size, tumor stage, tumor differentiation, lymph node metastasis, lymph node involvement rate, tumor invasion, metastasis, and survival time, were collected. Overall survival was defined as the time from treatment initiation to death. Notably, only 198 patients had complete overall survival information. This study was approved by the Clinical Research Ethics Committee (Approval No.202002054).

### 2.3 Tissue microarray construction

TMAs were constructed, and immunohistochemistry (IHC) was performed for B7H3 and HIF-1α. TMAs were prepared using an automated tissue array instrument. A 1 mm diameter tissue core was extracted from the tumor region of each paraffin-embedded specimen using a tissue microarrayer needle and precisely transferred to a pre-designed slide. Subsequently, 4 μm thick TMA sections were prepared.

### 2.4 Immunohistochemistry

Formalin-fixed, paraffin-embedded gastric cancer tissue microarrays were used for IHC. The paraffin sections were thoroughly dewaxed and dehydrated. The sections were then placed in a preheated pressure cooker with citrate buffer for antigen retrieval. After antigen retrieval at 121°C for 3 minutes, 3% hydrogen peroxide was added to block endogenous peroxidase activity. The sections were then incubated with 5% BSA blocking solution for 45 minutes. Subsequently, the sections were incubated overnight at 4°C with mouse anti-human B7H3 antibody (R&D Systems, MN, USA, #AF1027, 1:200) or rabbit anti-human HIF-1α antibody(R&D Systems, MN, USA, #MAB19352,Clone #2443C,1:200). Afterward, the sections were incubated with HRP-conjugated mouse secondary antibodies (Beyotime, Shanghai,China,#A0216) or rabbit secondary antibodies (Beyotime, Shanghai, China, #A0208) and observed under a microscope.

### 2.5 Evaluation of immunohistochemical staining

All IHC slides were independently and randomly reviewed, and scanned using the Dmetrix imaging system (Dmetrix, Tucson, AZ, USA). Slides were initially screened at low magnification (100x), and staining intensity was confirmed at higher magnification (400x). Each tumor core or peritumoral region was reviewed and scored. The percentage of positively stained cells was calculated as: (average number of positive cells/total number of cells from all fields) × 100. The histological staining scores for B7H3 and HIF-1α expression were graded as 0-3 (0: no staining; 1: <10% staining; 2: 10-50% staining; 3: >50% staining). Scores of 0 and 1 were classified as low expression, while scores of 2 and 3 were classified as high expression. Nuclear HIF-1α expression was scored using a binary system based on the percentage of tumor cells exhibiting nuclear staining. Cases with <10% nuclear-positive tumor cells were scored as 0 (negative for nuclear HIF-1α), while those with ≥10% nuclear-positive cells were scored as 1 (positive for nuclear HIF-1α).

### 2.6 Multiplex fluorescence immunohistochemistry

The TMA sections underwent the same steps as IHC, including dewaxing, dehydration, antigen retrieval, blocking, and incubation. The sections were incubated overnight at 4°C with rabbit anti-human HIF-1α antibody (R&D Systems, MN, USA, #MAB19352,Clone #2443C,1:200). After incubation, HRP-conjugated rabbit secondary antibody (Beyotime, Shanghai, China,#A0216) was added and incubated for 1 hour in the dark. The sections were then stained with TYR-570 fluorescent dye (AiFang biological, China, #AFIHC034) at room temperature for 10 minutes in the dark. After staining, the sections were subjected to antibody elution, followed by another round of antigen retrieval, blocking, and incubation. The sections were then incubated with mouse anti-human B7H3 antibody (R&D Systems, MN, USA, #AF1027, 1:200) at 4°C for 30 minutes in the dark, followed by HRP-conjugated mouse secondary antibody (Beyotime, Shanghai, China,#A0216) for 1 hour in the dark. The sections were then stained with TYR-520 fluorescent dye (AiFang biological, China, #AFIHC034) at room temperature for 10 minutes in the dark. Finally, the nuclei were visualized using DAPI staining, incubated for 10 minutes in the dark, and the sections were mounted and observed. Representative TMA images were acquired and analyzed using Mantra snap and Inform software (Akoya Biosciences).

### 2.7 Construction of stable B7H3 overexpression and knockdown gastric cancer cell lines

HGC-27 gastric cancer cells (Tong Pai Biological Technology Co. Ltd.) were infected with lentivirus carrying B7H3 knockdown (shRNA) or overexpression vectors (GenePharma, Suzhou, China). When the cells reached 70% confluency, puromycin was added for selection. Successfully transfected cells were subjected to limited dilution, and the cell suspension was serially diluted to a very low density until single cells were present in individual wells. The diluted cell suspension was then seeded into 96-well plates and cultured until single-cell colonies grew to cover 1/3 or 1/2 of the well area. Fifteen monoclonal cell lines were selected, expanded, and tested for B7H3 expression. Monoclonal cell lines confirmed to overexpress or knockdown B7H3 were cryopreserved for subsequent experiments.

### 2.8 Western blot analysis

Cells were washed with PBS and lysed using lysis buffer. After protein extraction, protein concentration was measured using a spectrophotometer. Proteins were then separated by SDS-PAGE (Beyotime, China, #P0012AC) and transferred to PVDF membranes (GE Healthcare Life Science, Germany). The membranes were blocked with 5% BSA (Fcmacs, Nanjing, China, #FMS-WB021) for 1 hour and incubated overnight at 4°C with mouse anti-human B7H3 antibody (R&D Systems, MN, USA, #AF1027, 1:1000) or rabbit anti-human HIF-1α antibody (Cell Signaling Technology,USA,#36169, Clone # D1S7W,1:1000). The next day, the membranes were incubated with HRP-conjugated mouse or rabbit secondary antibodies (Beyotime, Shanghai, China) at room temperature for 1 hour. Finally, the membranes were visualized using the ChemiDocTM MP imaging system (Bio-Rad) and ECL reagent (NCM Biotech, Suzhou, China, #10100).

### 2.9 Nuclear-cytoplasmic fractionation

B7H3-overexpressing and knockdown HGC-27 cells were treated with cobalt chloride to induce hypoxia. Nuclear and cytoplasmic proteins were extracted using a nuclear-cytoplasmic fractionation kit (Beyotime, Shanghai, China, #P0027) according to the manufacturer’s instructions.

### 2.10 Immunofluorescence

B7H3-knockdown HGC-27 cells were permeabilized with 0.1% Triton X-100 at room temperature for 15 minutes. The cells were then blocked with 5% BSA for 30 minutes, followed by incubation with rabbit anti-human HIF-1α antibody (Cell Signaling Technology, USA, #36169,Clone# D1S7W,1:800) at 4°C overnight. After incubation, HRP-conjugated rabbit secondary antibody (Beyotime, Shanghai, China, #A0208) was added and incubated for 1 hour in the dark. The cells were then stained with TYR-570 fluorescent dye (AiFang biological, China,#AFIHC034) at room temperature for 10 minutes in the dark. Finally, the nuclei were stained with DAPI, and the cells were observed and imaged.

### 2.11 Statistical analysis

Statistical analysis was performed using SPSS software (v19.0, IBM Corporation, Armonk, NY, USA). The chi-square test was employed to analyze differences in clinicopathological parameters among groups with varying expression levels of B7H3, HIF-1α, and nuclear HIF-1α. Spearman correlation analysis was used to assess the relationship between B7H3 and HIF-1α expression, as well as HIF-1α nuclear expression in gastric cancer. Survival analysis was performed using the Kaplan-Meier method. Univariate and multivariate Cox proportional hazards models were used to analyze prognostic factors in gastric cancer patients.

## 3 Results

### 3.1 High expression and correlation of B7H3 and HIF-1α in gastric cancer, and their combined high expression correlate with poor prognosis in patients

**Figure 1:**
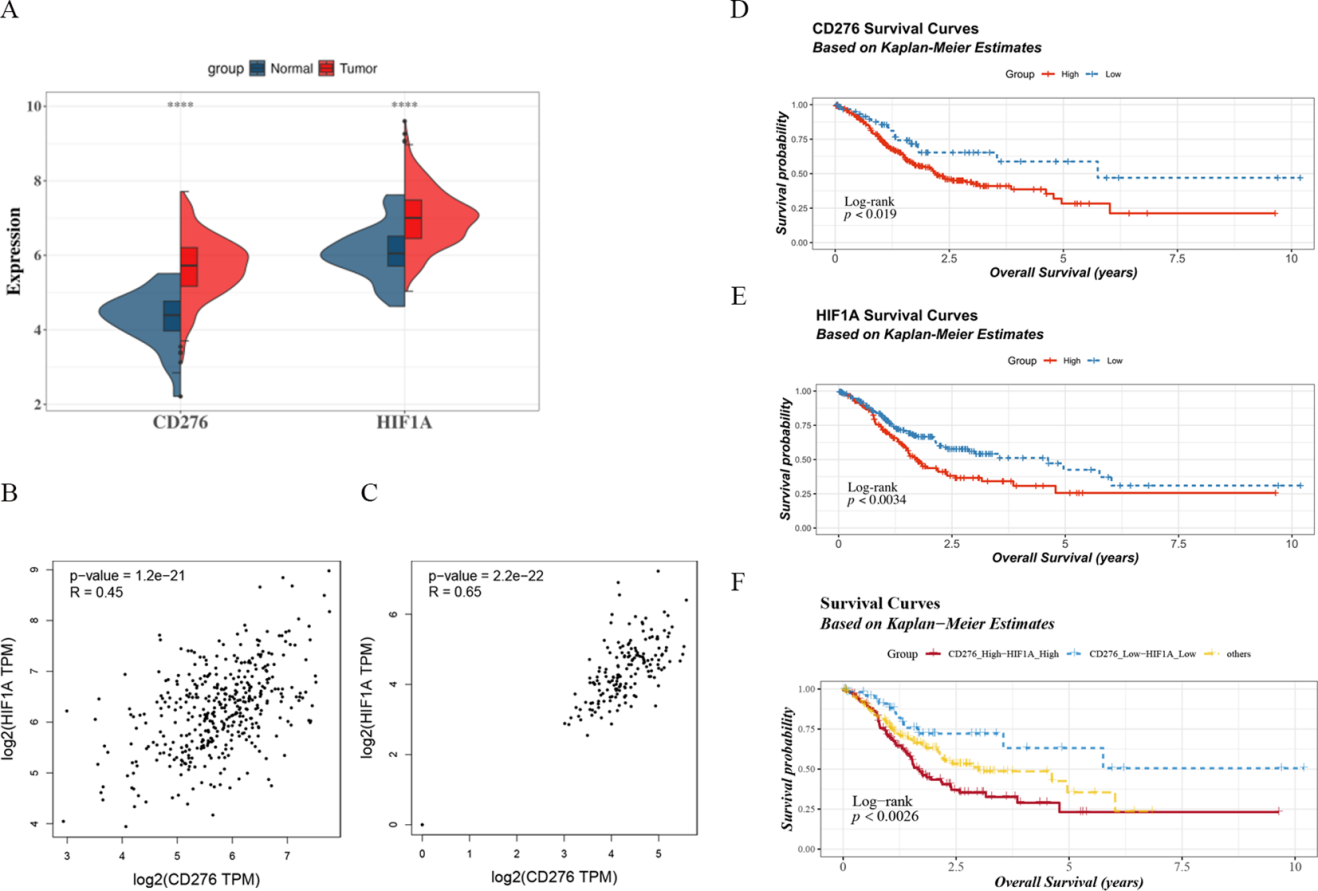
**(A)** Analysis of B7H3 and HIF-1α gene expression levels in gastric cancer (GC) and adjacent noncancerous tissues. **(B)** Analysis of the correlation between B7H3 and HIF-1α in gastric adenocarcinoma using the GEPIA database. **(C)** Analysis of the correlation between B7H3 and HIF-1α in adjacent noncancerous tissues using the GEPIA database. **(D)** Survival curve of patients with gastric cancer based on B7H3 expression. **(E)** Survival curve of patients with gastric cancer based on HIF-1α expression. **(F)** Survival curve of patients with gastric cancer based on combined B7H3 and HIF-1α expression. The red line indicates the high expression group, the blue line indicates the low expression group, and the yellow line indicates the other groups. **** indicates *P* < 0.001.

We analyzed expression data from 407 samples obtained from the UCSC database, and the results showed that both B7H3 and HIF-1α mRNA expression levels were significantly upregulated in gastric cancer tissues compared to adjacent noncancerous tissues (*P* < 0.001) (**Figure 1A**). we also evaluated B7H3 and HIF-1α expression in these tissues. These results showed a significant correlation between B7H3 and HIF-1α gene expression levels (r = 0.45, *P* = 1.2e−21) (**Figure 1B**). Additionally, analysis of adjacent noncancerous tissue samples revealed that B7H3 and HIF-1α expressions were also significantly correlated (r = 0.65, *P* = 2.2e−22) (**Figure 1C**). Notably, We investigated the impact of B7H3 and HIF-1α expression levels, as well as their combined expression, on the prognosis of patients with gastric cancer and found that the overall survival rate of patients with gastric cancer generally decreased, but the decline was more pronounced in patients with high expressions of B7H3 and HIF-1α (**Figures 1D, E**). Additionally, we divided the sample into three groups: those with high co-expression of B7H3 and HIF-1α, those with low co-expression, and others (B7H3 high and HIF-1α low, or B7H3 low and HIF-1α high). Patients with high co-expression of B7H3 and HIF-1α had the shortest survival period, and those with low co-expression had the best survival prognosis (*P* < 0.05) (**Figure 1F**).

### 3.2 The close association between B7H3 and HIF-1α expression in gastric cancer tissues

**Figure 2:**
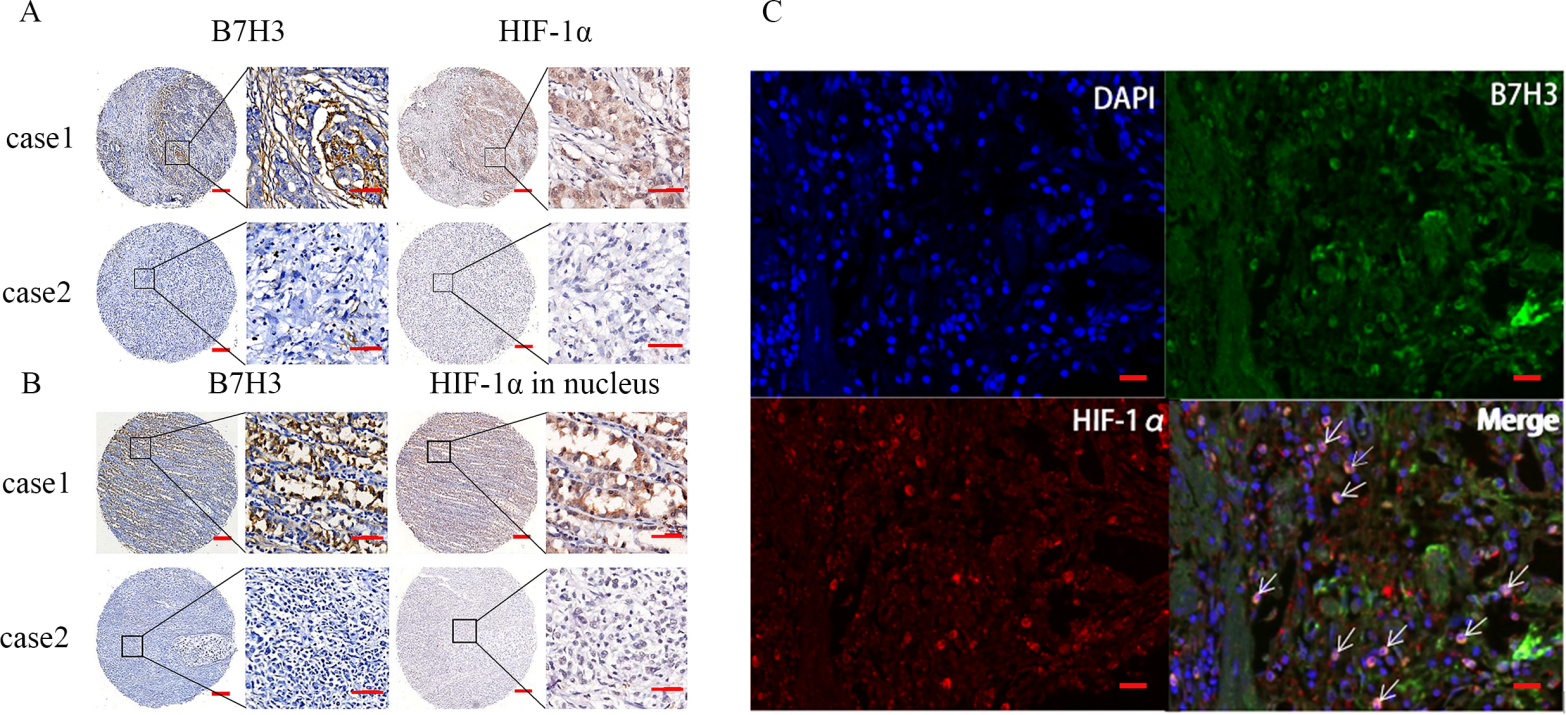
**(A)** Representative immunohistochemical (IHC) images of B7H3 and HIF-1α expression in gastric cancer tissues from 268 clinical patients with gastric cancer. **(B)** Representative IHC images of B7H3 and HIF-1α nuclear expression in gastric cancer tissues from 268 clinical patients with gastric cancer. Scale bars: 100 μm; 25 μm. **(C)** resentative multiplex fluorescence immunohistochemistry (mIHC) images of high B7H3 expression and HIF-1α nuclear expression in gastric cancer tissues. Scale bar: 100 μm.

To explore the correlation of the expression of B7H3 and HIF-1α in the gastric cancer tissues. we detected the expression of B7H3 and HIF-1α in 268 consecutive paraffin sections of gastric cancer tissues using immunohistochemistry. Representative images of B7H3 and HIF-1α expression in the same patient are shown in **Figure 2A**. We evaluated the proportion of cytoplasmic and nuclear staining in tumor cells and found that the spatial distribution of high B7H3 expression was consistent with that of high HIF-1α expression in gastric cancer tissues. However, statistical analysis revealed no significant correlation between B7H3 and HIF-1α protein expression (r_S_=0.070, *P* = 0.257) (**Table 1**). Additionally, we analyzed the nuclear expression of HIF-1α at different levels of B7H3 expression, as shown in the representative images in **Figure 2B**. The spatial distribution of B7H3 expression and HIF-1α nuclear expression here were also consistent. Moreover, We observed a moderate positive correlation between B7H3 expression and nuclear HIF-1α expression (r_S_ = 0.141, *P* = 0.021) (**Table 2**). Using multiplex fluorescence immunohistochemistry, we also localized the expression of B7H3 and HIF-1α in gastric cancer tissues and observed that high B7H3 expression was associated with increased nuclear expression of HIF-1α (**Figure 2C**).

**Table 1:** Correlation between B7H3 and HIF-1α expression.

B7H3 expression	HIF-1α expression		Spearman’s rank correlation	*P* value
	High	Low		
High	78	105	0.070	0.257
Low	30	55		

Correlation analysis of the staining index for B7H3 and HIF-1α protein expression levels in human gastric cancer specimens (n = 268).

**Table 2:** Correlation between B7H3 and HIF-1α nuclear expression.

B7H3 expression	HIF-1α nuclear expression		Spearman’s rank correlation	*P* value
	1	0		
High	103	80	0.141	***0.021***
Low	35	50		

Correlation analysis of the staining index for B7H3 and HIF-1α nuclear protein expression levels in human gastric cancer specimens (n = 268), where HIF-1α nuclear expression is categorized into two groups (1 represents nuclear expression; 0 represents no nuclear expression). Statistical significance is indicated by bold italic P values < 0.05.

### 3.3 The relationship between B7H3 and HIF-1α expression, pathological parameters, and prognosis in gastric cancer patients

**Figure 3:**
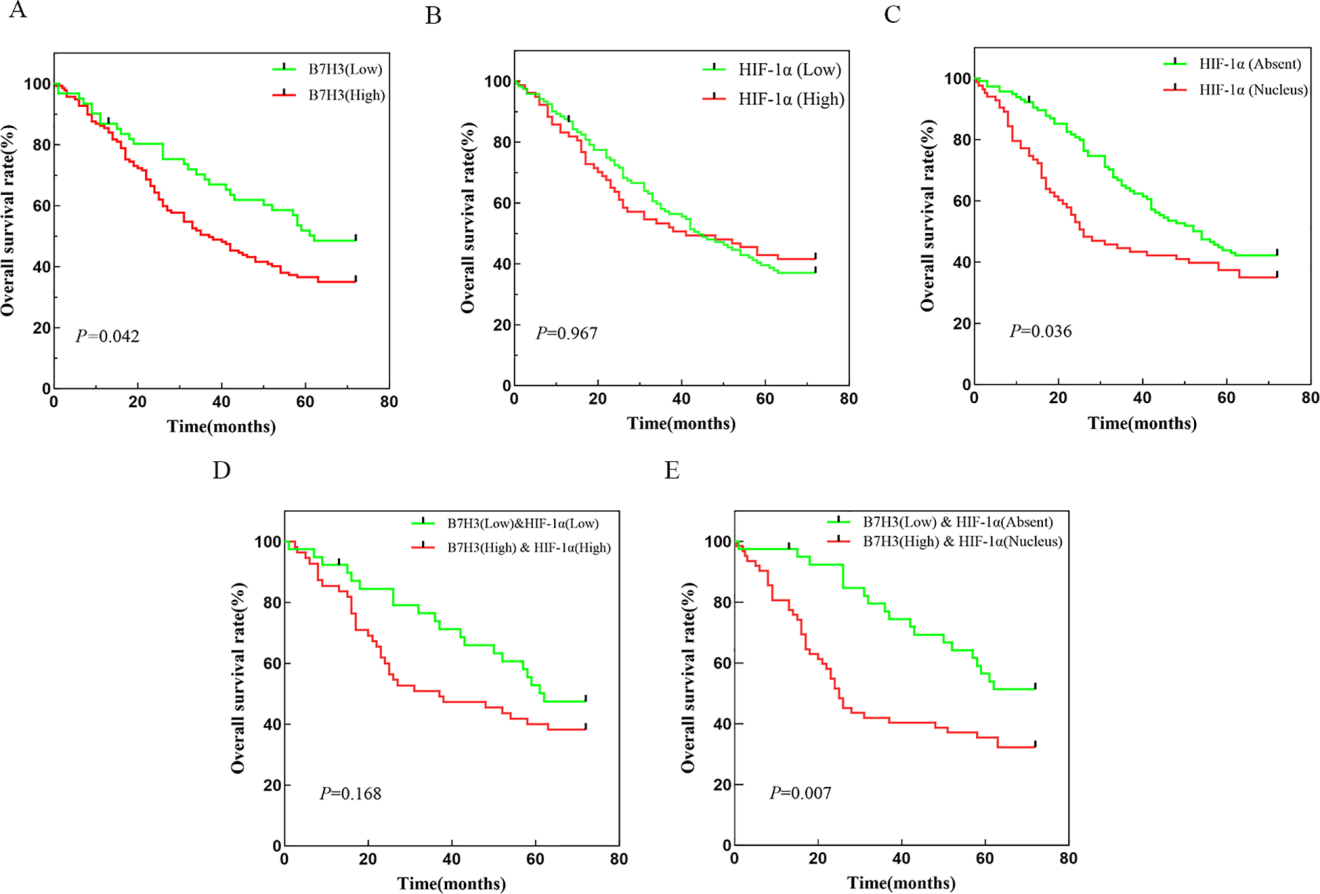
**(A)** Survival curve of gastric cancer patients based on B7H3 expression levels. **(B)** Survival curve of patients with gastric cancer based on HIF-1α expression levels. **(C)** Survival curve of patients with gastric cancer based on whether HIF-1α was expressed in the nucleus. **(D)** Survival curve of patients with gastric cancer based on the co-expression of B7H3 and HIF-1α. **(E)** Survival curve of patients with gastric cancer based on the co-expression of B7H3 and nuclear HIF-1α.

We organized the clinical pathological data of 268 patients with gastric cancer and combined it with the expression levels of B7H3 and HIF-1α in tissue microarray (TMA) samples in order to explore the impact of B7H3 and HIF-1α on the clinical prognosis of patients with gastric cancer. Results showed that B7H3 expression was closely associated with tumor staging (*P* < 0.001), lymph node involvement (*P* = 0.047), and depth of tumor invasion (*P* < 0.001) (**Table 3**). Additionally, high B7H3 expression was associated with shorter overall survival (*P* = 0.042) (**Figure 3A**). Although we did not find a significant correlation between HIF-1α expression and clinical pathological parameters in patients with gastric cancer (**Table 3**), nor did HIF-1α expression levels affect overall survival (**Figure 3B**), nuclear expression of HIF-1α was significantly associated with tumor size (*P* < 0.001) and lymph node metastasis staging (*P* = 0.002) (**Table 3**), and patients with nuclear HIF-1α expression had poorer survival (*P* < 0.001) (**Figure 3C**). Furthermore, we also analyzed the correlation between the co-expression of B7H3 and HIF-1α and clinical pathological features in patients with gastric cancer and found that high co-expression of B7H3 and HIF-1α was positively correlated with tumor staging (*P* = 0.008) and depth of tumor invasion (*P* = 0.003) (**Table 3**). Although patients with high co-expression of B7H3 and HIF-1α had shorter survival times, the difference was not statistically significant (*P* = 0.168) (**Figure 3D**). High B7H3 expression combined with nuclear HIF-1α expression was associated with higher tumor size (*P* < 0.001), tumor staging (*P* = 0.001), lymph node metastasis (*P* = 0.021), lymph node metastasis staging (*P* = 0.001), and depth of tumor invasion (*P* = 0.002) (**Table 3**). Moreover, high B7H3 expression combined with nuclear HIF-1α expression was significantly associated with poorer overall survival (*P* = 0.007) (**Figure 3E**).

**Table 3:** The relationship between B7H3 and HIF-1α expression and clinical pathological features in gastric cancer patients.

Clinical Pathological Features	B7H3 Expression		*P* value	HIF-1α Expression		*P* value	HIF-1α Nuclear Expression		*P* value	B7H3 and HIF-1α Co-Expression				*P* value	B7H3 and HIF-1α Nuclear Co-Expression			*P* value
	High	Low		High	Low		1	0		0	1	2	3		0	1	2	
Gender			0.611			0.911			0.528					0.625				0.915
Female	38	20		23	35		32	26		11	9	24	14		10	26	22	
Male	145	65		85	125	106	104	44	21	81	64	40	89	81
Age			0.281			0.211			0.255					0.135				0.142
<70	116	48		71	93		89	75		29	19	64	52		23	77	64	
≥70	67	37		37	67	49	55	26	11	41	26	27	38	39
Tumor Size			0.155			0.990			***<0.001***					0.223				***<0.001***
<5cm^3^	122	64		75	111		24	106		39	25	72	50		41	88	57	
≥5cm^3^	61	21		33	49	58	80	16	5	33	28	9	27	46
Tumor Staging			***<0.001***			0.398			0.103					***0.008***				***0.001***
0	3	8		7	4		7	4		3	5	1	2		2	8	1	
Ⅰ	19	13		14	18	12	20	7	6	11	8	9	15	8
Ⅱ	40	26		26	40	31	35	15	11	25	15	16	29	21
Ⅲ	94	29		44	79	64	59	25	4	54	40	18	52	53
Ⅳ	27	9		17	19	24	12	5	4	14	13	5	11	20
Tumor Differentiation			0.083			0.715			0.218					0.186				0.794
Well-differentiated	52	4		57	84		77	64		32	20	52	37		2	2	2	
Moderately differentiated	31	90		50	71	59	62	21	10	50	40	20	53	48
Poorly differentiated	2	89		1	5	2	4	2	0	3	1	28	60	53
Lymph Node Metastasis			***0.047***			0.318			0.131					0.211				***0.021***
N0	51	34		38	47		38	47		16	18	31	20		19	43	23	
N1-3	132	51		70	113	100	83	39	12	74	58	31	72	80
Lymph Node Metastasis Staging			0.091			0.301			***0.002***					0.325				***0.001***
N0	51	34		38	47		38	47		16	18	31	20		19	43	23	
N1	43	19		21	41	24	38	15	4	26	17	14	29	19
N2	43	13		29	27		33	23		10	3	17	26		9	18	29	
N3	46	19		20	45	43	22	14	5	31	15	8	25	32
Depth of tumor invasion			***<0.001***			0.458			0.330					***0.003***				***0.002***
Ⅰ	17	19		16	20		17	19		12	7	8	9		10	18	8	
Ⅱ	18	16		16	18	13	21	8	8	10	8	10	17	7
Ⅲ	124	45		64	105	94	75	32	13	73	51	25	70	74
Ⅳ	24	5		12	17	14	15	3	2	14	10	5	10	14
Distant Metastasis			0.530			0.252			0.170					0.289				0.180
M0	161	77		93	145		119	119		51	26	94	67		46	104	88	
M1	22	8		15	15	19	11	4	4	11	11	4	11	15

Correlation between the high and low expression of B7H3 and HIF-1α, as well as their co-expression, and clinical pathological parameters in gastric cancer patients. B7H3 expression: 0 (low expression), 1 (high expression). HIF-1α expression: 0 (low expression), 1 (high expression). HIF-1α nuclear expression: 0 (nonnuclear expression), 1 (nuclear expression). Co-expression of B7H3 and HIF-1α: 0 (both low expression), 1 (B7H3 high expression and HIF-1α low expression), 2 (B7H3 low expression and HIF-1α high expression), 3 (both high expression). Co-expression of B7H3 and HIF-1α nuclear expression: 0 (B7H3 low expression and HIF-1α nonnuclear), 1 (B7H3 high expression and HIF-1α nonnuclear or B7H3 low expression and HIF-1α nuclear), 3 (B7H3 high expression and HIF-1α nuclear). Statistical significance is indicated by bold italic *P* values < 0.05.

### 3.4 High B7H3 expression and HIF-1α nuclear expression as independent prognostic risk factors for gastric cancer patients

We evaluated the risk factors associated with the prognoses of patients with gastric cancer and found that, in univariate analysis, high B7H3 expression, HIF-1α nuclear expression, and their combined high expression were high-risk factors for poor prognosis (*P* < 0.05) (**Table 4**). Notably, when high B7H3 expression, HIF-1α nuclear expression, tumor size, lymph node metastasis, and distant metastasis were included in a multivariate analysis of prognostic factors, HIF-1α nuclear expression was an important independent prognostic factor for overall of patients with gastric cancer (*P* < 0.05) (**Table 5**).

**Table 4:** Univariate Cox proportional hazards analysis for overall survival in gastric cancer patients.

Factors	Univariate analysis	
	HR (95%CI)	*P* value
Gender (Female vs. Male)	0.901 (0.583-1.391)	0.638
Age (<70 vs. ≥70)	1.203 (0.839-1.724)	0.315
Tumor Size (<5cm^3^ vs. ≥5cm^3^)	1.829 (1.267-2.640)	***0.001***
Lymph Node Metastasis (N0 vs. N1-3	3.381 (2.106-5.426)	***<0.001***
Distant Metastasis (No vs. Yes)	2.118 (1.278-3.509)	***0.004***
B7H3 Expression (Low vs. High)	1.519 (1.009-2.288)	***0.045***
HIF-1α Expression (Low vs. High)	0.992 (0.686-1.436)	0.968
HIF-1α Nuclear Expression (No vs. Yes)	1.820(1.267-2.614)	***0.001***
Co-expression of B7H3 and HIF-1α (No vs. Yes)	0.852 (0.594-1.211)	0.383
Co-expression of B7H3 and HIF-1α Nuclear Expression (Others vs. High Expression and Nuclear)	1.775 (1.237-2.545)	***0.002***

Statistical significance is indicated by bold italic P values < 0.05.

**Table 5:** Multivariate Cox proportional hazard analysis for overall survival in gastric cancer patients.

Factors	HR(95%CI)	*P* value
Tumor Size (<5cm^3^ vs. ≥5cm^3^)	1.320(0.888-1.962)	0.169
Lymph Node Involvement (N0 vs. N1-3)	3.062(1.883-4.979)	***<0.001***
Distant Metastasis (No vs. Yes)	1.574(0.941-2.631)	0.084
HIF-1α Nuclear Expression (No vs. Yes)	1.607(1.078-2.397)	***0.020***
B7H3 Expression (Low vs. High)	1.149(0.750-1.761)	0.523

Statistical significance is indicated by bold italic P values < 0.05.

### 3.5 B7H3 upregulates HIF-1α protein expression and nuclear localization in gastric cancer cells

**Figure 4:**
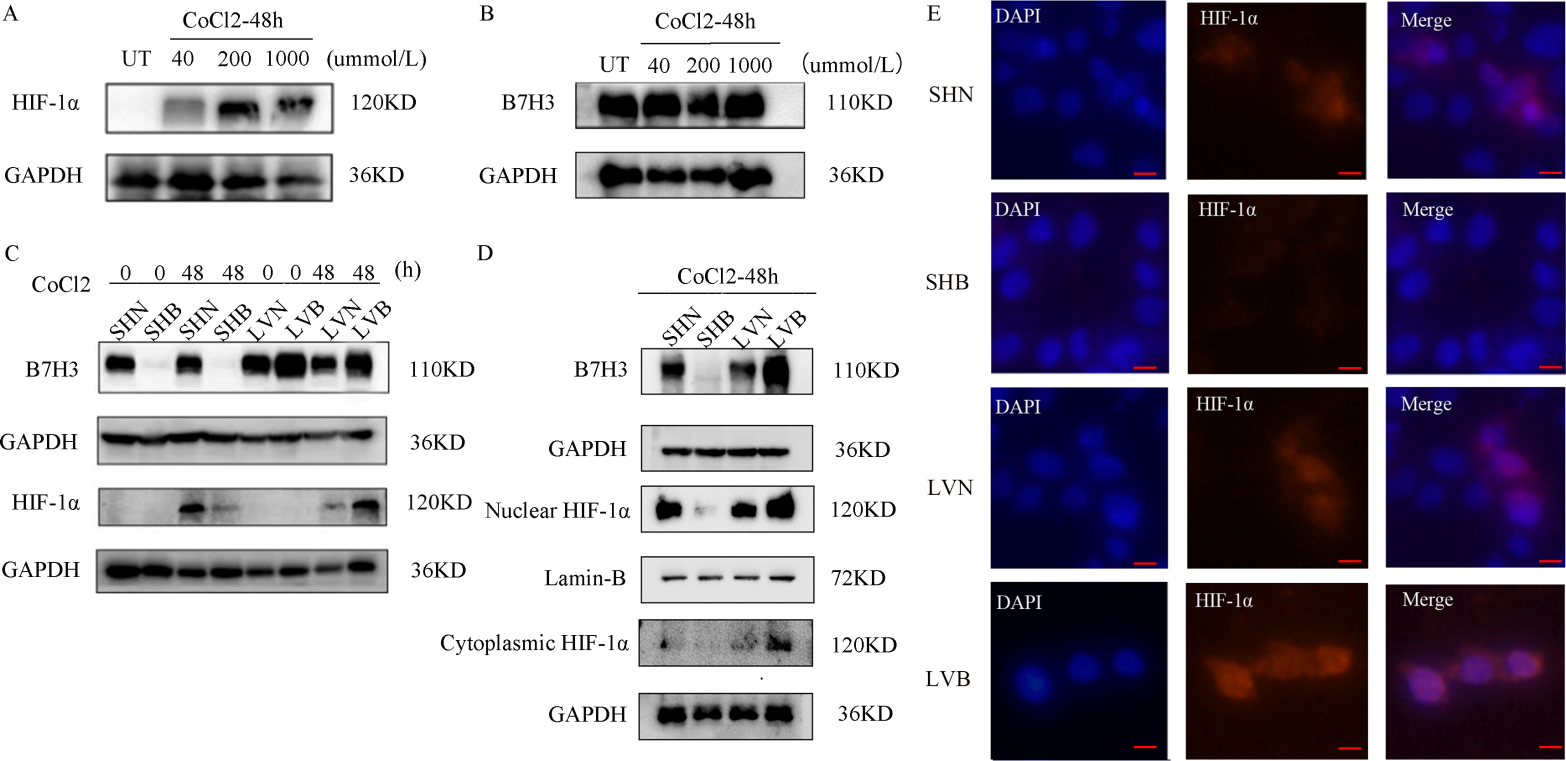
**(A)** HIF-1α protein expression in HGC-27 cells under gradient concentrations of cobalt chloride induction. **(B)** B7H3 protein expression in HGC-27 cells under gradient concentrations of cobalt chloride (40 μmol/L, 200 μmol/L, 1000 μmol/L) induction. **(C)** HIF-1α protein expression in HGC-27 cells with overexpressed or knocked-down B7H3 under hypoxic and normal conditions. **(D)** HIF-1α expression in nuclear and cytoplasmic proteins of HGC-27 cells with overexpressed or knocked-down B7H3 under cobalt chloride induction. **(E)** Fluorescence immunohistochemistry of HGC-27 cells with B7H3 knockdown (SHB),B7H3 overexpression (LVB) and control group (SHN/LVN). Blue fluorescence represents DAPI, and red fluorescence represents HIF-1α. Scale bar: 10 μm.

To investigate the regulatory relationship between B7H3 and HIF-1α protein levels in gastric cancer, we constructed a cellular hypoxia model using cobalt chloride. We found that the 200 μmol/L cobalt chloride induced the best hypoxic effect (**Figure 4A**). To study the effect of HIF-1α expression on B7H3 expression in gastric cancer cells, we detected B7H3 protein expression in HGC-27 cells after induction with different concentrations of cobalt chloride for 48 hours. Compared to the control group, there was no significant difference in B7H3 protein expression regardless of the concentration of cobalt chloride used (**Figure 4B**). However, we also induced these cells with 200 μmol/L cobalt chloride for 48 hours by constructing monoclonal cell lines with stable overexpression and knockdown of B7H3, which showed that overexpression of B7H3 significantly upregulated HIF-1α protein expression and that knockdown of B7H3 significantly downregulated HIF-1α expression (**Figure 4C**).

Next, we performed nuclear-cytoplasmic separation on cobalt chloride-induced B7H3 overexpressing and knockdown HGC-27 cells and found that overexpression of B7H3 significantly increased HIF-1α expression in nuclear proteins, whereas stable knockdown of B7H3 significantly reduced HIF-1α expression in nuclear proteins (**Figure 4D**). Additionally, we found that overexpression of B7H3 significantly increased HIF-1α expression in cytoplasmic proteins compared to the control group, but knockdown of B7H3 did not result in significant changes in HIF-1α expression (**Figure 4D**). Furthermore, using multicolor immunofluorescence, we observed that overexpression of B7H3 significantly increased the nuclear expression of HIF-1α in HGC-27 cells, whereas stable knockdown of B7H3 significantly reduced the nuclear expression of HIF-1α (**Figure 4E**).

## 4 Discussion

This study revealed that B7H3 and HIF-1α nuclear expression are moderately positively correlated in gastric cancer and that high B7H3 expression combined with HIF-1α nuclear expression can serve as an indicator of poor prognosis in gastric cancer. Additionally, B7H3 can promote the protein expression and nuclear localization of HIF-1α in gastric cancer cells. These findings suggest that developing combined therapies to target B7H3 and HIF-1α may be a promising new strategy for treating gastric cancer.

Under hypoxic conditions, the stability and transcriptional activity of HIF-1α are significantly increased, and upon activation HIF-1α is translocated to the tumor cell nucleus, where it leads to the transcriptional activation or repression of downstream target genes related to metabolism, inflammation, vascular homeostasis, and tumor development (25). Consistent with previous studies, we found that B7H3 and HIF-1α expressions were significantly increased in gastric cancer compared to adjacent noncancerous tissues (26, 27). Furthermore, we found a significant correlation between B7H3 and HIF-1α gene expressions. However, in gastric cancer tissues, although HIF-1α and B7H3 expressions showed consistent spatial distribution, their association does not reach statistical significance. This may mean that the protein expressions of B7H3 and HIF-1α are not synchronized with their mRNA expressions, or the result could be due to limitations in sample size and scoring errors.

We also found that high B7H3 expression and HIF-1α nuclear expression were significantly associated with clinical pathological features in gastric cancer patients, such as tumor staging, depth of tumor invasion, lymph node metastasis, and survival prognosis. When B7H3 was highly expressed and HIF-1α was co-expressed in the nucleus, the risks of tumor growth, invasion, and metastasis were significantly increased, and the survival prognosis of patients with gastric cancer was poorer. Several studies have shown that B7H3 not only promotes the occurrence, development, metastasis, and resistance of gastric cancer but is also associated with poor prognosis in gastric cancer (27, 28). Additionally, the level of HIF-1α nuclear expression can influence tumor size, lymph node metastasis, and prognosis in gastric patients (29, 30). Given that B7H3 and HIF-1α have similar roles in the development of gastric cancer, combining our experimental results suggests that when B7H3 is highly expressed and HIF-1α is co-expressed in the nucleus, there may be an additive effect on promoting gastric cancer development and metastasis.

Related studies have shown that B7H3 can promote the stability of HIF-1α by increasing ROS in breast cancer (31) and may enhance HIF-1α expression by upregulating NF-κB phosphorylation levels in lung cancer (24). Similarly, we found that B7H3 can promote HIF-1α protein expression in gastric cancer tissues. Notably, we also found that B7H3 is associated with HIF-1α nuclear expression in gastric cancer tissues and that B7H3 can promote the nuclear localization of HIF-1α. Currently, there is a lack of research on the relationship between B7H3 and HIF-1α nuclear expression, and the mechanism by which B7H3 regulates HIF-1α nuclear localization remains unclear. Several cytokines and metabolic enzymes within the tumor microenvironment, such as IL-6 (32), CXCR-4 (33), fatty acid synthase (34), have been shown to promote HIF-1α nuclear translocation. Notably, B7-H3 has been widely reported to be closely associated with the production of these factors and enzymes in various diseases (35–37). Therefore, B7-H3 may potentially regulate the nuclear translocation of HIF-1α by modulating the secretion of IL-6, FASN, and CXCR-4, although the precise underlying molecular mechanisms remain to be further elucidated. The regulatory role of B7-H3 in HIF-1α nuclear translocation expands our understanding of its functional mechanisms in modulating the tumor microenvironment, while also providing a novel theoretical foundation for the clinical application of B7-H3-targeted antibodies.

Although monoclonal antibodies targeting B7H3 have entered clinical trials, they still have relatively low response rates and resistance in clinical applications. However, bispecific antibodies targeting B7H3, such as B7H3 combined with PD-L1 or CD3, have achieved certain success in anti-tumor applications (38, 39). Based on the importance of the hypoxic tumor microenvironment in tumor immune evasion and tolerance (40), targeting both B7H3 and HIF-1α may regulate the hypoxic pathway by inhibiting HIF-1α expression, alter the immunosuppressive tumor microenvironment, reduce drug resistance, and enhance anti-tumor effects, thereby improving the efficacy of B7H3 immunotherapy and achieving synergistic effects. Therefore, the results of this study suggest that the combined detection of B7H3 and HIF-1α nuclear expression can serve as a new method for predicting gastric cancer prognosis and provide evidence in support of the development of B7H3-combined-with-HIF-1α dual-targeted therapy.

## Data Availability

Publicly available datasets were analyzed in this study. This data can be found here: https://xenabrowser.net/datapages/?dataset=TCGA-STAD.sampleMap%2FSTAD_HTSeq_FPKM&host=https%3A%2F%2Ftcga.xenahubs.net(TCGA-STAD). http://gepia.cancer-pku.cn/(GEPIA).
